# Comparative Study on the Mechanism of Macrophage Activation Induced by Polysaccharides from Fresh and Dried Longan

**DOI:** 10.3390/nu16111654

**Published:** 2024-05-28

**Authors:** Shengwei Wang, Xiaoyan Chen, Qianxin Li, Yinghui Zhang, Yu Rong, Yanxian Feng, Hui Liu, Jucai Xu, Ruili Yang, Wu Li

**Affiliations:** 1 Guangdong Provincial Key Laboratory of Large Animal Models for Biomedicine, School of Pharmacy and Food Engineering, Wuyi University, Jiangmen 529020, China; 2 Key Laboratory of Food Nutrition and Functional Food of Hainan Province, College of Food Science and Engineering, Hainan University, Haikou 570228, China; 3 College of Food Science, South China Agricultural University, Guangzhou 510642, China

**Keywords:** longan polysaccharide, macrophage activation, RNA-seq, NF-κB pathway, MAPK pathway

## Abstract

Longan (*Dimcarpus longan* Lour.) is a kind of traditional fruit used as a medicine and a food. Fresh longan is primarily consumed as a fruit, whereas dried longan is commonly employed for medicinal purposes. The differences in the immunomodulatory activities and mechanisms of polysaccharides between dried and fresh longan remain unclear. The present study comparatively analyzed the mechanisms of macrophage activation induced by polysaccharides from dried (LPG) and fresh longan (LPX). The results revealed that LPG and LPX differentially promoted macrophage phagocytosis and the secretion of NO, TNF-α, and IL-6. RNA-seq analysis revealed that LPG and LPX differentially affected gene expression in macrophages. The LPG treatment identified *Tnf* and chemokine-related genes as core genes, while *myd88* and interferon-related genes were the core genes affected by LPX. A comprehensive analysis of the differentially expressed genes showed that LPG initiated macrophage activation primarily through the TLR2/4-mediated *TRAM/TRAF6* and CLR-mediated *Src/Raf1* NF-κB signaling pathways. LPX initiated macrophage activation predominantly via the CLR-mediated *Bcl10/MALT1* and NLR-mediated *Rip2/TAK1* MAPK and NF-κB signaling pathways. Interestingly, the non-classical NF-κB signaling pathway was activated by polysaccharides in both dried and fresh longan to elicit a slow, mild immune response. LPG tends to promote immune cell migration to engage in the immune response, while LPX facilitates antigen presentation to promote T cell activation. These findings contribute insights into the mechanisms underlying the differences in bioactivity between dried and fresh longan and their potential applications in immune-enhancing strategies and functional-food development.

## 1. Introduction

Polysaccharides are essential macromolecules that significantly contribute to the growth and vigor of living organisms [1]. Pharmacological studies have shown that numerous natural polysaccharides exhibit notable biological and pharmacological activities, including anticoagulant [2], anti-tumor [3], antidiabetic [4], and prebiotic activities [5] and immunomodulatory properties [6]. Owing to their safety and non-toxicity, certain bioactive polysaccharides have been widely used in functional foods and clinical applications. For instance, lentinan [7], *Ganoderma lucidum* polysaccharide [8], and *Astragalus* polysaccharides have been formulated into pharmaceutical products [9].

In recent decades, despite the extensive research conducted on the structure and activity of polysaccharides derived from natural sources, the correlation between polysaccharide structure and activity remains enigmatic. Research has shown that different sources, species, and processing methods can influence polysaccharides’ structure, biological activity, and mechanisms [10]. For example, fungus polysaccharides from different genera showed notable variations in their digestive properties and anti-inflammatory activity [11]. The molecular weight, monosaccharide composition, and antioxidant capacity of loquat leaf polysaccharides were significantly influenced by varying drying methods [12]. The drying process enhances the branching structure of longan polysaccharides and affects their immunomodulatory properties [13]. However, the mechanism behind the differences in the activity of polysaccharides from different sources and species or that have undergone different processing methods remain perplexing.

Longan (*Dimcarpus longan* Lour.) is a kind of traditional fruit used as a medicine and a food. Fresh longan is primarily served as a fruit, whereas dried longan is commonly employed for medicinal purposes [14]. Previous studies have shown that the immunomodulatory activities of polysaccharides from dried and fresh longan are performed via different receptor-mediated signaling pathways. Specifically, dried longan-derived polysaccharides engage Toll-like receptors (TLRs) 2/4 and the Ca^2+^/CR3 receptor, whereas fresh longan-derived polysaccharides primarily act through TLR2/4 receptors. Both dried and fresh longan-derived polysaccharides have been reported to exert immunomodulatory effects via the *MyD88/TRAF6*-mediated MAPK and PI3K/AKT signaling pathways [15,16,17]. However, due to the fragmented results of previous research, it remains unclear whether other receptors and signaling pathways are involved in the immunomodulatory activities of polysaccharides from dried and fresh longan. Additionally, a comprehensive understanding of the differences in their activities and mechanisms of action has yet to be established.

Therefore, we compared the effects of polysaccharides from dried (LPG) and fresh longan (LPX) on macrophage activation. Additionally, the effects on gene expression were comparatively analyzed. This study may contribute to improving our understanding of the immunomodulatory mechanisms of polysaccharides in both dried and fresh longan and their potential functional-food application.

## 2. Materials and Methods

### 2.1. Materials and Chemicals

DMEM medium and fetal bovine serum (FBS) were obtained from GIBCO (Thermo Fisher, Waltham, MA, USA). TLR4 inhibitor (TAK-242), neutral red, and lipopolysaccharide (LPS) were obtained from Sigma (St. Louis, MO, USA). DAF-FM DA was purchased from Beyotime (Shanghai, China). Anti-TLR2 antibodies (TLR2-IN-C29) were obtained from Abcam (ab285439, Cambridge, MA, USA). Mouse TNF-α kit and IL-6 kits were procured from R&D Systems (Minneapolis, MN, USA).

### 2.2. Polysaccharides

The polysaccharides from both dried and fresh longan were obtained in our previous study [13]. The average molecular weights of LPG and LPX were 1.06 × 10^7^ g/mol and 1.31 × 10^7^ g/mol, respectively. LPG consists of rhamnose, mannose, glucose, and galactose in a molar ratio of 0.2:0.21:1:0.2. LPX is composed of mannose and glucose in a molar ratio of 0.59:1. The major glycosidic bond of LPG were →6)-α-d-Glcp-(1→, →3)-β-d-Glcp-(1→, →3)-β-d-Galp-(1→ and →3)-α-l-Rhap-(1→. The major glycosidic bond of LPX was →6)-α-d-Glcp-(1→. Endotoxin was not detected in LPG or LPX.

### 2.3. Cell Culture

RAW 264.7 cells were acquired from the cell bank of the Chinese Academy of Sciences (Shanghai, China). The cells were cultivated in DMEM medium containing 10% FBS and incubated in a humidified incubator at 37 °C with 5% CO_2_.

### 2.4. Macrophage Phagocytosis

Phagocytic activity was assessed by employing a neutral red assay [18]. RAW 264.7 cells were inoculated in a 96-well plate (2 × 10^5^ cells/mL) for 24 h at 37 °C. LPG and LPX were used to stimulate RAW 264.7 cells under the same concentrations (final concentrations: 25, 50, 100, and 200 μg/mL). The positive control consisted of LPS at a concentration of 5 μg/mL, while the negative control consisted of the medium alone. The supernatants were discarded, and 100 μL of 0.1% neutral red solution was added after incubation for 24 h. After 20 min culture, 200 μL of cell lysis buffer (100% ethanol and 0.1 M acetic acid in a ratio of 1:1) was added to the washed cells and incubated for 4 h at 4 °C. Microplate readers were used for measuring at 540 nm. The results are presented as a percentage of the control.

### 2.5. NO, TNF-α, and IL-6 Content Detection

RAW 264.7 cells were treated with LPG and LPX (final concentrations: 25, 50, 100, and 200 μg/mL, respectively) at a density of 2 × 10^5^ per well. The positive control was established using LPS at a concentration of 5 μg/mL, while the negative control consisted of DMEM. After incubation for 12 h, the supernatants were collected to measure TNF-α and IL-6 levels, and the cells were incubated with DAF-FM DA for 30 min to determine NO levels, by using a commercial kit following the manufacturer’s guidelines.

### 2.6. Effects of Toll-like Receptor Inhibitors on Macrophages

RAW 264.7 was preincubated with TLR2, TLR4 inhibitors alone, and in combination for 30 min. Upon removal of the supernatant, LPG and LPX at terminal concentration of 100 μg/mL were added and incubated for 24 h to measure the NO, TNF-α, and IL-6 levels.

### 2.7. Transcriptome Sequencing

Total RNA was extracted from RAW 264.7 cells treated with LPG and LPX using TRIzol^®^ Reagent (Thermo Fisher, Waltham, MA, USA). The quality of RNA was assessed using a 5300 Bioanalyzer (Agilent, Palo Alto, CA, USA), and the quantity was measured with an ND-2000 instrument (ThermoFisher Scientific, Waltham, MA, USA). The cDNA library was prepared using the Illumina^®^ Stranded mRNA Prep Ligation Kit from Illumina (San Diego, CA, USA) and sequenced with the Illumina HiSeq 2500 by RIBOBIO Biotechnology Co., Ltd. (Guangzhou, China).

### 2.8. Bioinformatics Analysis

After obtaining clean data, DESeq2 (version 1.24.0) was used to detect differentially expressed genes (DEGs), which were defined as |log2 fold change| > 1 and *p-adjust* < 0.05. The PCA analysis, volcano plots creation, and cluster analysis of DEGs were conducted using R language (version 4.2.2). Gene Ontology (GO) and Kyoto Encyclopedia of Genes and Genomes (KEGG) analysis were employed for functional annotation and signaling pathway analysis of DEGs. String database and Cytoscape were applied to identify gene interaction networks. Pathview package in R was used to visualize pathways.

### 2.9. Statistical Analysis

Data are reported as mean ± standard error of mean (SEM) with a minimum of three independent experiments. Comparison of differences among multiple groups was performed using one-way ANOVA, followed by Tukey’s post hoc test. The statistical analysis used SPSS 26.0 software (SPSS Inc., Chicago, IL, USA). Statistical significance was determined at *p* < 0.05. Graphs were built by GraphPad Prism9 software (GraphPad, Bethesda, MD, USA).

## 3. Results

### 3.1. Effects on Phagocytosis

**Figure 1:**
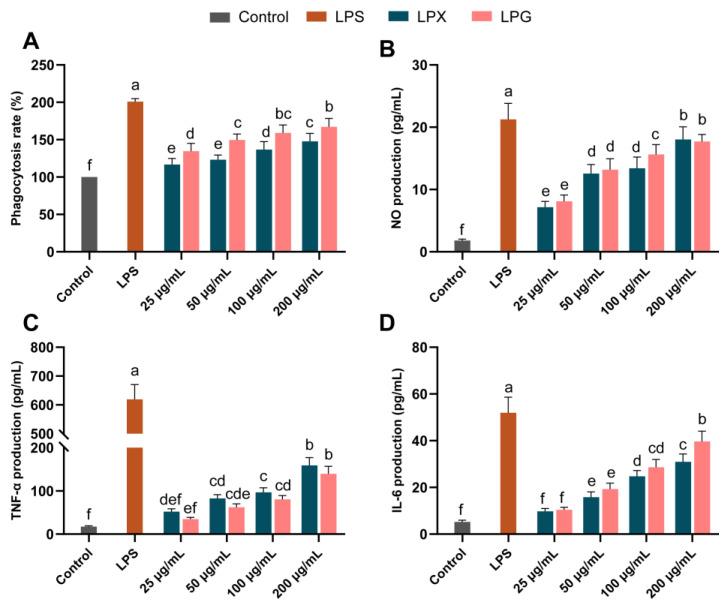
Effects of LPG and LPX on the phagocytosis (**A**), NO production (**B**), TNF-α (**C**), and IL-6 (**D**) secretion of macrophages. The presented values denote means ± SEM, *n* = 5. Significant differences (*p* < 0.05) are indicated by different letters.

Figure 1A depicts the impact of LPG and LPX on macrophage phagocytosis. A dose-dependent enhancement in macrophage phagocytosis by LPG and LPX was observed at 25−200 μg/mL. Phagocytosis rates were increased to 167.25% and 147.91% of those of the control group by 200 μg/mL LPG and LPX treatment, respectively. The results showed that LPG treatment induced a stronger phagocytic effect on macrophages compared to LPX (*p* < 0.05).

### 3.2. Effects on NO Production

As shown in Figure 1B, LPG and LPX treatment led to a remarkable concentration-dependent increase in NO levels. At 100 μg/mL, the LPG group had significantly higher NO levels than the LPX group. At 200 μg/mL, the NO levels were 9.91- and 9.74-fold higher than in the control group for LPG and LPX treatment, respectively, with no significant difference between them (*p* > 0.05).

### 3.3. Effects on TNF-α and IL-6 Secretion

As displayed in Figure 1C,D, treatment with LPG and LPX (25−200 μg/mL) induced a significant increase in TNF-α and IL-6 in a dose-dependent manner. At 200 μg/mL, the TNF-α levels in the LPG and LPX groups were 7.93- and 9.02-fold that of the control group, respectively. The IL-6 levels in the LPG and LPX groups were 7.53- and 5.87-fold higher compared to the control group, respectively. In the concentration range of 25−200 μg/mL, there was no significant difference in TNF-α secretion between the LPG and LPX groups (*p* > 0.05). At 200 μg/mL, the LPG group showed a significant increase in IL-6 secretion compared to the LPX group (*p* < 0.05). The above results indicate that both LPG and LPX could activate macrophages, although they exhibit some differences in function, and the molecular details should be further investigated to unravel the differences in the molecular mechanisms of macrophage regulation by LPG and LPX.

### 3.4. Effects of LPG and LPX on Gene Expression

**Figure 2:**
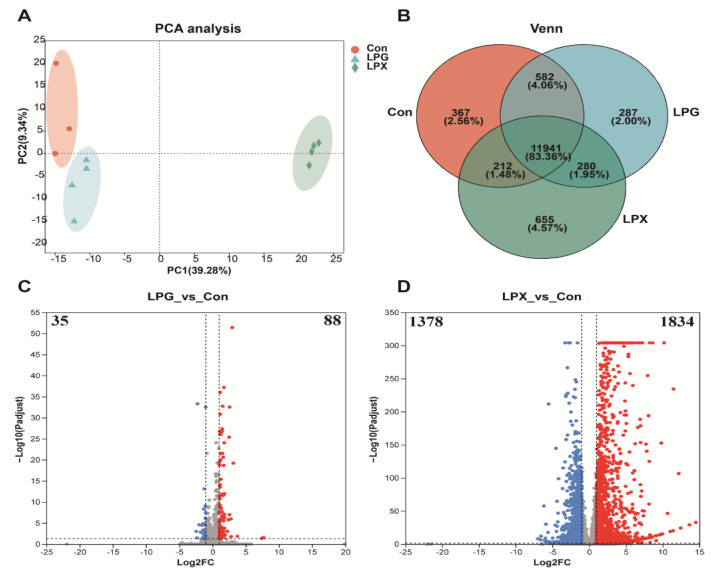
Changes in gene expression. (**A**) PCA analysis; (**B**) Venn analysis. (**C**,**D**) display volcano plots of differentially expressed genes in LPG (left) and LPX (right) groups, respectively. Red dots indicate upregulated gene expression (log2 fold change > 2), blue dots indicate downregulated gene expression (log2 fold change ≤ 2), and gray dots indicate non-significant differences.

Principal Component Analysis (PCA) and Venn diagram analysis were employed to comprehensively discern the impact of LPG and LPX treatment on gene expression levels. PCA analysis (Figure 2A) revealed three well-distinguished groups, indicating different gene expression patterns between the LPG and LPX groups. The Venn diagram analysis (Figure 2B) indicated that 287 genes and 655 genes were uniquely expressed in the LPG group and LPX group, respectively. The above results demonstrate that the addition of LPG and LPX differentially affects RAW 264.7 gene expression.

### 3.5. Differentially Expressed mRNAs

The volcano plots in Figure 2C,D reflect the DEGs for the LPG and LPX treatments, respectively. As shown in Figure 2C, LPG treatment resulted in 123 DEGs, with 88 genes up-regulated and 35 genes down-regulated. LPX treatment resulted in 3212 DEGs (Figure 2D), with 1834 genes up-regulated and 1378 genes down-regulated. The up-regulated expressed genes accounted for 71.54% and 57.10% of DEGs in the LPG and LPX groups, respectively.

### 3.6. Functional Prediction of DEGs

**Figure 3:**
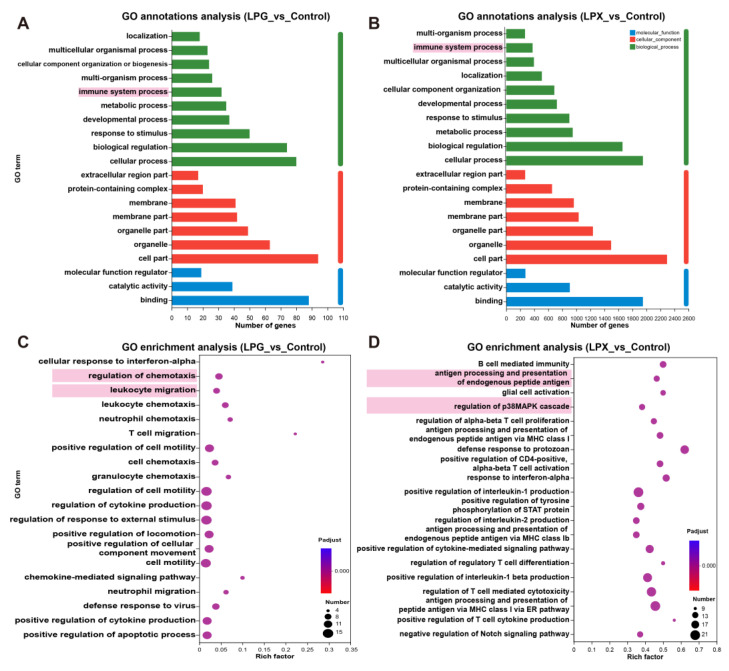
GO annotation and enrichment analysis of LPG and LPX groups. (**A**,**B**) GO annotation analysis of the LPG and LPX groups, respectively. Biological processes (green), cellular components (red) and molecular functions (blue). Terms annotated with immune functions are highlighted with a pink background. (**C**,**D**) GO enrichment analysis of the LPG and LPX groups, respectively. The size of the circles represents the number of enriched genes. Representative terms are marked with a pink background.

GO annotation was conducted to further clarify the functional roles of DEGs. As shown in Figure 3A,B, the most annotated items for genes in the LPG and LPX groups included binding, cellular fraction, and immune system processes, indicating that both LPG and LPX affect the expression of immune-related genes. Further GO enrichment analysis (Figure 3C) showed that DEGs in the LPG group were significantly enriched in cytokine production and chemotaxis in immune cells. For the LPX group (Figure 3D), DEGs were significantly enriched in p38 MAPK cascade regulation and terms associated with antigen presentation and T cell activation. These results suggest that LPG primarily affects the chemotaxis and cytokine secretion functions of macrophages, whereas LPX predominantly impacts the antigen-presentation function of macrophages.

### 3.7. Signaling Pathway Analysis of DEGs

**Figure 4:**
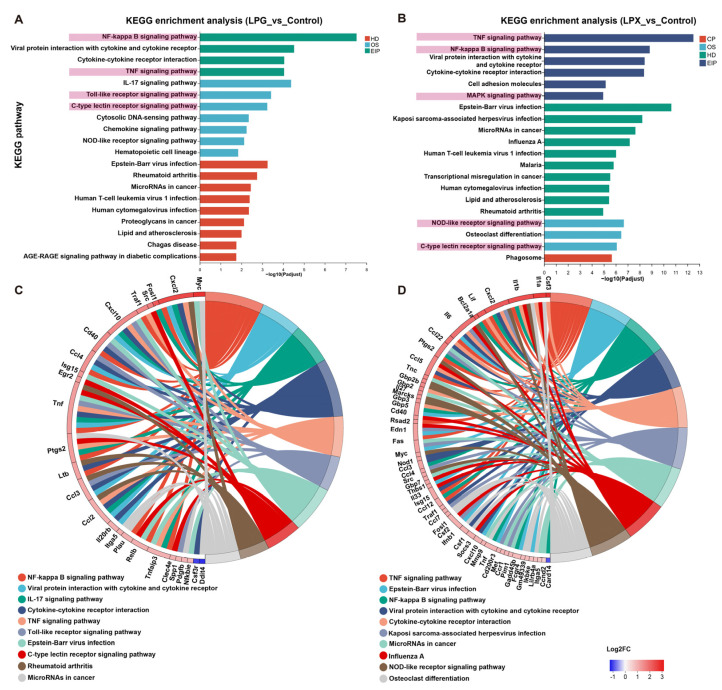
KEGG pathway analysis and enriched chordograms. (**A**,**B**) KEGG enrichment analysis of the LPG and LPX groups, respectively. Terms associated with receptors and signaling pathways are highlighted with pink background. (**C**,**D**) Enrichment chordograms for the LPG and LPX groups, respectively.

The significant KEGG pathways between the LPG and control groups are shown in Figure 4A. The DEGs of the LPG group were mainly enriched in the “NF-kappa B (NF-κB) signaling pathway”, “Toll-like receptor signaling pathway”, “C-type lectin receptor (CLR) signaling pathway”, and “TNF signaling pathway”. The significantly enriched pathways in the LPX group (Figure 4B) included the “TNF signaling pathway”, “NF-kappa B signaling pathway”, “MAPK signaling pathway”, “NOD-like receptor (NLR) signaling pathway”, and “C-type lectin receptor signaling pathway”. The relationship between the major DEGs and the top 10 pathways are exhibited in Figure 4C,D. The major DEGs enriched in the CLR signaling pathway of both the LPG and LPX groups are shown in Figure S1. These results suggest that the main activity mechanisms of LPG were the TLR- and the CLR-mediated downstream NF-κB signaling pathways. The main activity mechanisms of LPX were the NLR- and the CLR-mediated downstream MAPK and NF-κB pathways. Additionally, the involvement of cytokine and TNF-related pathways in the activation of macrophages by LPG and LPX is of significant importance.

### 3.8. Core Gene Analysis

**Figure 5:**
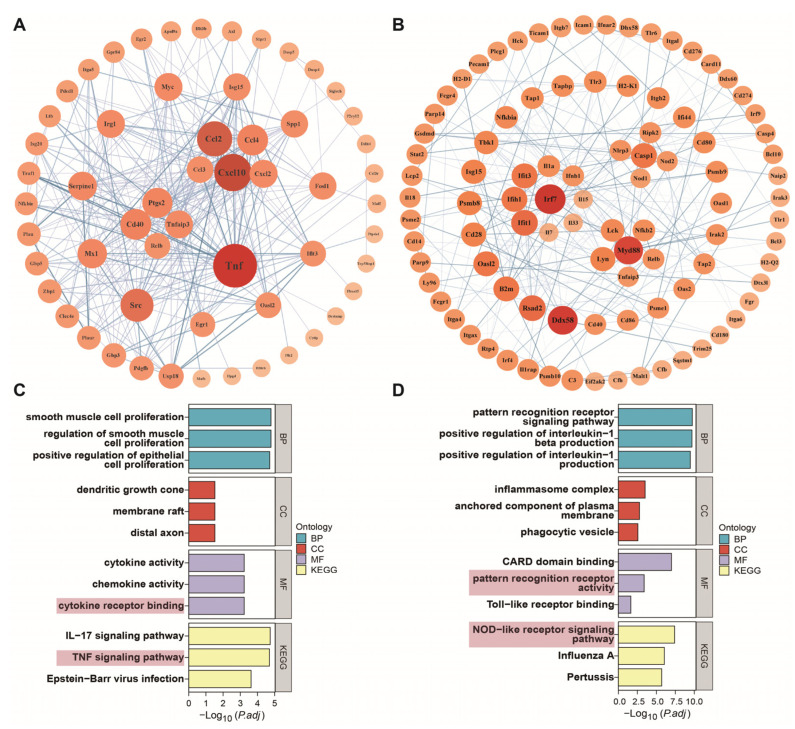
Core gene analysis of DEGs. Genetic interaction network of LPG (**A**) and LPX (**B**) groups. The area and color of the circles represent the degree of the genes. Core gene enrichment analysis of LPG (**C**) and LPX (**D**) groups. Terms which are significantly enriched in MF categories and KEGG are highlighted with pink background.

The genetic interaction network of the LPG group is shown in Figure 5A. *Tnf* and *cxcl10* were identified as the primary focus in the interaction network consisting of 57 genes. These genes displayed a significant degree of connectivity, with *Tnf* exhibiting 37 edges and *cxcl10* possessing 28 edges, underscoring their critical roles as central hubs within the network. Chemokines and NF-κB pathway-related genes also play crucial regulatory roles, which is consistent with the KEGG enrichment results. Activated macrophages are involved in the immune reaction through the secretion of several cytokines, including TNF-α and interleukin, as well as chemokines [19]. For the LPX group (Figure 5B), *Myd88* and *Irf7* emerged as the primary genes of interest within a network comprising 95 genes. These genes displayed a significant degree of connectivity, with *Myd88* having 14 edges and *Irf7* having 16 edges. These results further demonstrate that the NF-κB and MAPK pathways are activated by LPX.

### 3.9. Effects of TLR Inhibitor on Macrophage Activation

**Figure 6:**
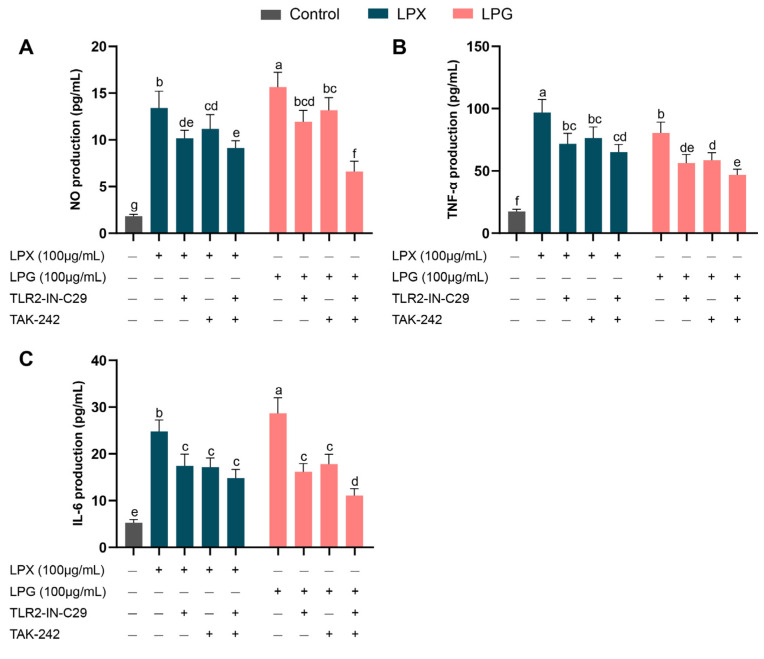
Effects of TLR inhibitors on macrophage NO (**A**), TNF-α (**B**), and IL-6 (**C**) secretion. The presented values denote means ± SEM, *n* = 5. Significant differences (*p* < 0.05) are indicated by different letters.

It was reported that TLRs are extensively involved in the activation of immune cells by longan polysaccharides [16]. Thus, we comparatively analyzed the role of TLRs in macrophage activation by LPG and LPX, employing TLR inhibitors. As depicted in Figure 6, the exposure of RAW 264.7 cells to the medium alone resulted in the minimal production of NO and IL-6. Compared to the LPG treatment, pre-incubation with TLR2 or TLR4 inhibitors alone significantly reduced NO and IL-6 production, and the combined use of TLR2 and TLR4 inhibitors showed stronger attenuation (*p* < 0.05). This may be attributed to the formation of a TLR2 and TLR4 heterodimer, which cooperatively mediates the activation of longan polysaccharides [20]. In addition, compared to the LPX group, the combined TLR inhibitors showed a more pronounced inhibitory effect on the cytokine secretion induced by LPG treatment. This finding aligns with the KEGG signaling pathway analysis, indicating that TLR plays a crucial role in macrophage activation induced by LPG. The activation of macrophages by LPX may be primarily mediated by other receptors.

## 4. Discussion

Macrophages are the primary effector cells of intestinal mucosal immunity, capable of sensing intestinal antigens and participating in intestinal immune responses [21,22]. Previous studies have indicated that longan polysaccharides can reach the intestine in an undigested form without significant changes in immunomodulatory activity [6]. Longan polysaccharides may exert immunomodulatory activity by activating macrophages within the intestinal immune system [23]. The present study showed that polysaccharides extracted from dried and fresh longan differentially promote phagocytosis and NO, TNF-α, and IL-6 secretion. Phagocytosis, as one of the important symbols of macrophage activation, plays an essential role in macrophage phagocytosis and antigen clearance. IL-6 is a pivotal regulator of the macrophage immune response, promoting B cell antibody secretion and T cell growth [24]. TNF-α is another marker of macrophage activation and activates multiple immune-related signaling pathways. Additionally, TNF-α could promote the expression of *c-myc* and *c-fos*, which are closely associated with cell proliferation [25].

GO enrichment analysis revealed that LPG treatment primarily impacted cytokine production and macrophage chemotactic function. In contrast, LPX treatment primarily influenced the activation of the macrophage MAPK signaling pathway and the function of antigen presentation to T cells. Macrophages’ chemotactic function is a reflection of their ability to migrate and localize to the site of infection or injury. This enables them to direct the migration and colonization of immune and repair cells, facilitating the clearance of pathogens and tissue repair [26,27]. The antigen presentation function of macrophages plays a pivotal role in activating and expanding T cells, thereby promoting the immune response, regulating immune homeostasis, and facilitating the development of immune memory [28,29]. These findings suggest that LPG and LPX may play different roles in immune regulation. LPG promotes the secretion of chemokines and cytokines, which could facilitate the recruitment and attraction of various immune cells to migrate and congregate, consequently contributing to the immune response and injury repair. Meanwhile, LPX may induce macrophages to capture, process, and display antigenic fragments more efficiently, thereby enhancing T cell activation and the immune response. These functional distinctions have important implications for the application of LPG and LPX in immune disease remission, immune-enhancing strategies, and functional-food applications.

A series of core genes, including *Tnf*, *Cxcl10*, *Ccl2*, *Src*, *Myc*, and *Tnfaip3*, were identified in the LPG group (Figure 5A). *Ccl2* and *Cxcl10* are typical pro-inflammatory chemokines exerting chemotactic effects on migrating macrophages [30,31]. *c-Myc* is a crucial transcription factor functioning in the cell cycle and cell proliferation and serves as one of the targets for anti-tumor strategies [32,33]. *Src* can promote cell proliferation by activating p38 [34]. *TNF* can bind to the TNFR1 receptor and up-regulate *TRAF2/5* expression, further activating the NF-κB pathway and eliciting an immune cascade response [35]. But the overexpression of *TNF* may cause cytotoxic effects [36]. Interestingly, the expression of *Tnfaip3*, a negative regulator of the NF-κB signaling pathway [37], occurs concurrently, potentially exerting the inhibitory effects on NF-κB overactivation to mitigate inflammation [38]. This may be one of the reasons for the capacity of longan polysaccharide to elicit a controlled immune response as a functional food.

*Myd88*, *Irf7*, *Ddx58*, *Nod1*, *Nod2*, *Nlrp3*, and *Casp1* were identified as core genes in the LPX group (Figure 5B). *Myd88* is a key junctional molecule that mediates the activation of downstream NF-κB and MAPK signaling pathways [39]. *IRF7* belongs to the interferon regulatory factor family capable of activating the downstream JAK-STAT signaling pathway, promoting the expression of type I interferon genes, and activating the immune response [40]. *DDX58* encodes the RIG-I protein and is associated with anti-tumor immune response [41]. Recent research has elucidated the involvement of *DDX58* in the immune response to COVID-19 [42]. Interestingly, the “Coronavirus disease—COVID-19” pathway was also significantly enriched in immunity-associated genes based on GO annotation (Figure S2). This hints at a potential contribution of longan polysaccharides in combating COVID-19. *Nod1* and *Nod 2* are members of the NLR family which can recognize peptidoglycan fragments, and promote NF-κB activation and cytokine production [43]. *NLRP3* acts as a sensor protein for inflammatory vesicles, activating *caspase-1* (*casp1*) and triggering the release of pro-inflammatory cytokines [44]. Although both LPG and LPX are capable of activating macrophages, the core genes affected by LPG and LPX are distinctly different. Combined with core gene enrichment analysis (Figure 5C,D), LPG mainly affected TNF-α-related genes and signaling pathways to elicit an immune cascade response, while LPX mainly affected NLR- and interferon-related genes and signaling pathways for the immune cascade response.

In addition, *Relb* was discovered as a core gene in both the LPG and LPX groups. As a major member of the non-classical NF-κB signaling pathway, *Relb* assumes a significant role in many biological processes, including lymphatic organ development, immune cell maturation, and the response [45]. The classical NF-κB signaling pathway activates the IKK complex, leading to the degradation of the IκB protein. This process also leads to the release of the NF-κB/Rel complex, which regulates the activity of p50/RelA and p50/c-Rel heterodimers [46]. Unlike the classical NF-κB pathway, *p100* is degraded to form active *p52* through the actions of NF-κB-induced kinase (NIK) and *IκBα*. This results in the formation of the RelB/p52 complex, which is conveyed into the nucleus to initiate the expression of target genes [47]. The non-classical NF-κB pathway is mainly reliant on extracellular signaling molecules, including *LTB*, *Cd40*, and *BAFF*. These molecules lead to the kinase-mediated degradation of *TRAF3* and activate the signaling pathway. In the present study, Both *Cd40*, *LTB*, and *RelB* were found to be significantly up-regulated in the LPG and LPX groups (Figure S3), demonstrating the activation of the non-classical NF-κB pathway. This is distinct from previous findings of longan polysaccharides and other natural polysaccharides that primarily focused on the classical NF-κB signaling pathway [48]. Actually, the non-classical NF-κB signaling pathway allows for relatively slow and sustained activation of the NF-κB signaling pathway [49]. This characteristic is crucial in preventing excessive acute immune responses and aligns better with the mode of action of natural products.

**Figure 7:**
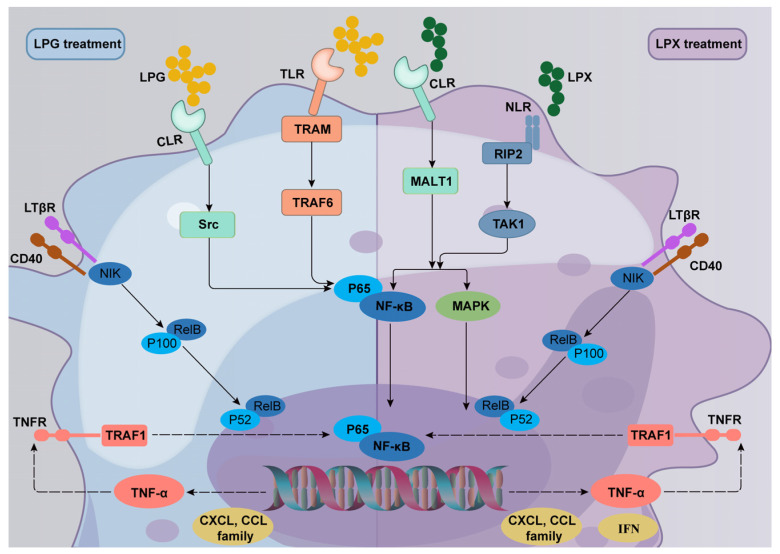
Molecular mechanisms underlying the activation of macrophages by LPG and LPX.

The combination of the DEGs, KEGG enrichment analysis, and TLR inhibitor results indicated that LPG primarily activates macrophages through the TLR2/4-mediated *TRAM/TRAF6* and CLR-mediated *Src/Raf1* NF-κB signaling pathways. In contrast, LPX predominantly activates macrophages through the CLR-mediated *Bcl10/MALT1* and NLR-mediated *Rip2/TAK1* MAPK and NF-κB signaling pathways. The main mechanisms of macrophage activation by LPG and LPX can be summarized as follows: (a) LPG binds to TLR and CLR to activate the downstream NF-κB signaling pathway, promoting the secretion of chemokines and TNF-α, and facilitating the up-regulation of *CD40* expression. (b) TNF-α binds to *TNFR*, further amplifying the immune response, while up-regulating the expression of the negative regulator *Tnfaip3* to avoid an inflammatory response. (c) The up-regulated expression of *CD40* further activates the non-classical NF-κB signaling pathway to exert a delayed and sustained immune response. Distinct from the LPG group, LPX mainly activates the downstream NF-κB and MAPK signaling pathways through CLR and NLR. Meanwhile, the activation of NLR promotes the secretion of interferons. Furthermore, TNF-α and *CD40* facilitate the amplification of the immune response and activation of the non-classical NF-κB pathway. The mechanism of macrophage activation by LPG and LPX is demonstrated in Figure 7. Additionally, compared to previous studies, the mechanism of dried longan-derived polysaccharide action is further confirmed. However, fresh longan polysaccharides were found to activate macrophages through the NF-κB and MAPK signaling pathways, and the involvement of the non-canonical NF-κB pathway was also elucidated.

## 5. Conclusions

In conclusion, our results demonstrate that LPG and LPX differentially affected gene expression in macrophages, resulting in differential effects on macrophage activation. LPG initiates macrophage activation mainly through the TLR2/4-mediated *TRAM/TRAF6* and CLR-mediated *Src/Raf1* NF-κB signaling pathways. LPX initiates macrophage activation predominantly via the CLR-mediated *Bcl10/MALT1* and NLR-mediated *Rip2/TAK1* MAPK and NF-κB signaling pathways. The TNF-α signaling pathway mediates further immune cascade responses, and the non-classical NF-κB signaling pathway is also activated to elicit a slow, mild immune response.

## Data Availability

The data presented in this study are openly available in the NCBI database (BioProject: PRJNA996753).

## Supplementary Materials

The following supporting information can be downloaded at: https://www.mdpi.com/article/10.3390/nu16111654/s1, Figure S1: The major DEGs in the CLR signaling pathway of the LPG (A) and LPX (B) groups; Figure S2: KEGG enrichment analysis of immune-related gene sets in LPG (A) and LPX (B) groups based on GO annotation; Figure S3: Heatmap of DEG expression enriched in NF-κB signaling pathway of LPG (A) and LPX groups (B).
