# Supplementary material for: Comparative Study on the Mechanism of Macrophage Activation Induced by Polysaccharides from Fresh and Dried Longan
Source: Nutrients. 2024 May 28;16(11):1654. doi: 10.3390/nu16111654 (PMC11174042; doi:10.3390/nu16111654)
Supplement: Supplementary file 1 [file nutrients-16-01654-s001.zip › nutrients-2999430-supplementary.pdf]

Supplementary data

Figure captions

Figure S1. The major DEGs in the CLR signaling pathway of the LPG (A) and LPX groups (B).

Figure S2. KEGG enrichment analysis of immune-related gene sets in LPG (A) and LPX (B) groups based on GO annotation.

Figure S3. Heatmap of DEGs expression enriched in NF-κB signaling pathway of LPG (A) and LPX groups (B).

Figure S1

A

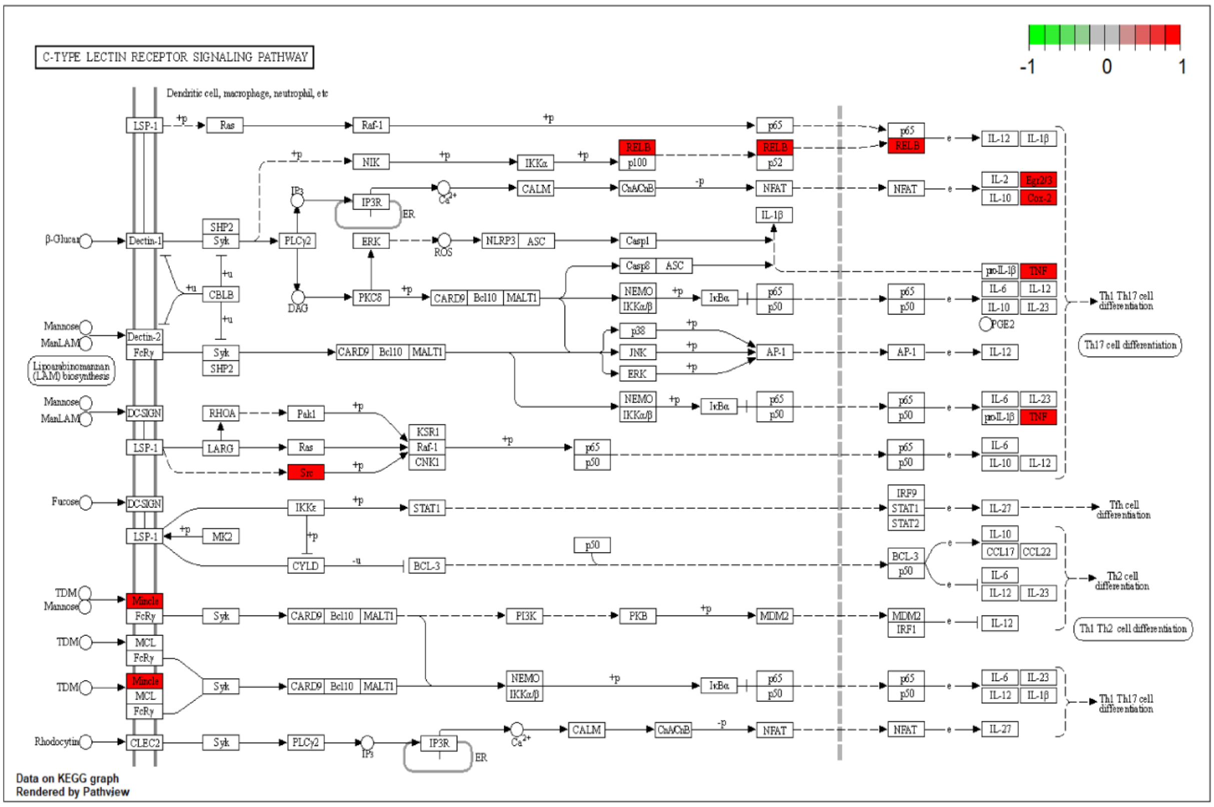
C-TYPE LECTIN RECEPTOR SIGNALING PATHWAY
-1
0
1
Dendritic cell, macrophage, neutrophil, etc
LSP-1
Ras
Raf-1
p65
RELB
NIK
IKKα
p100
p52
CALM
CnA/CnB
NFAT
IL-1β
β-Glucan
Dectin-1
SHP2
Syk
PLCγ2
IP3R
ER
ERK
ROS
NLRP3
ASC
Casp1
Casp8
CBLB
DAG
PKCδ
CARD9
Bcl10
MALT1
NEMO
IKKα/β
IκBα
p50
Mannose
ManLAM
Dectin-2
FcRγ
Lipoarabinomannan (LAM) biosynthesis
p38
JNK
AP-1
DCSIGN
RHOA
Pak1
KSR1
CNK1
LARG
Src
Fucose
IKKε
STAT1
MK2
CYLD
BCL-3
IRF9
STAT2
TDM
Mincle
PI3K
PKB
MDM2
IRF1
MCL
Rhodocytin
CLEC2
IL-12
IL-2
Egr2/3
IL-10
Cox-2
pro-IL-1β
TNF
IL-6
IL-23
PGE2
IL-27
CCL17
CCL22
Th1 Th17 cell differentiation
Th17 cell differentiation
Tfh cell differentiation
Th2 cell differentiation
Th1 Th2 cell differentiation
Data on KEGG graph
Rendered by Pathview

B

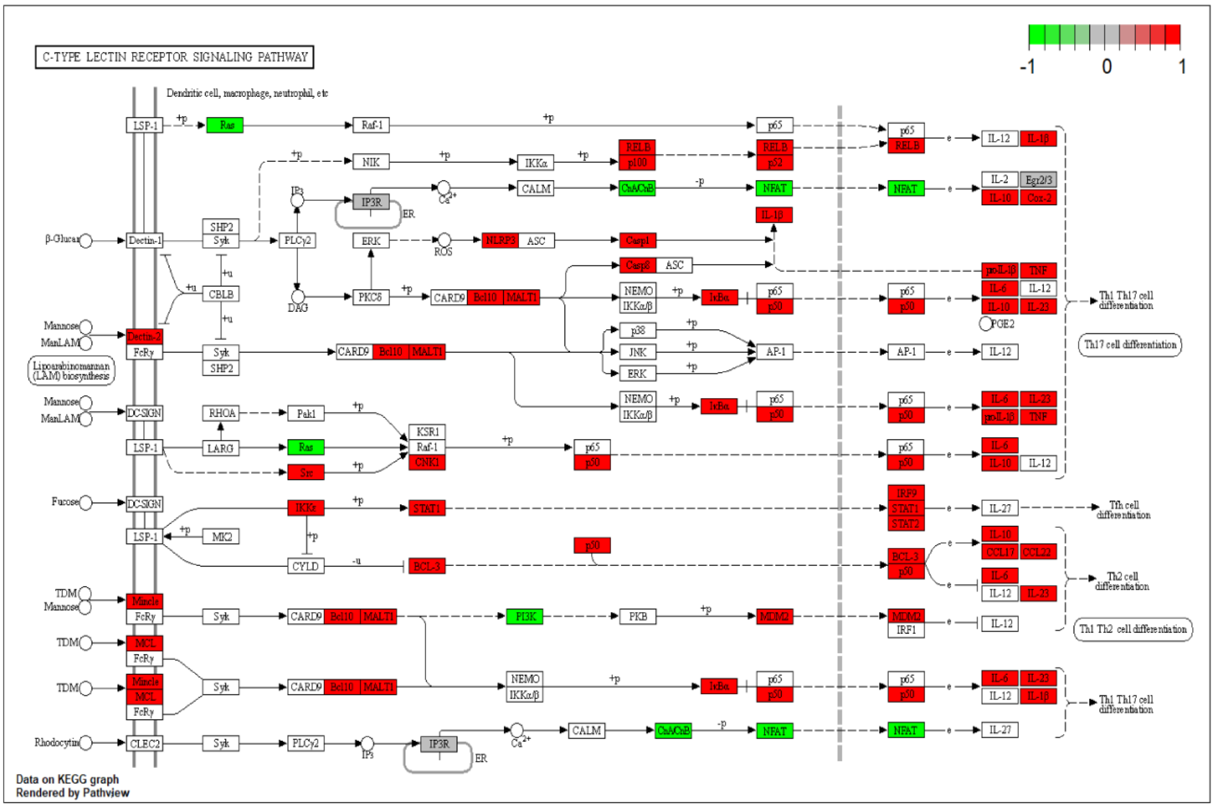
C-TYPE LECTIN RECEPTOR SIGNALING PATHWAY
-1
0
1
Dendritic cell, macrophage, neutrophil, etc
Data on KEGG graph
Rendered by Pathview

Figure S2

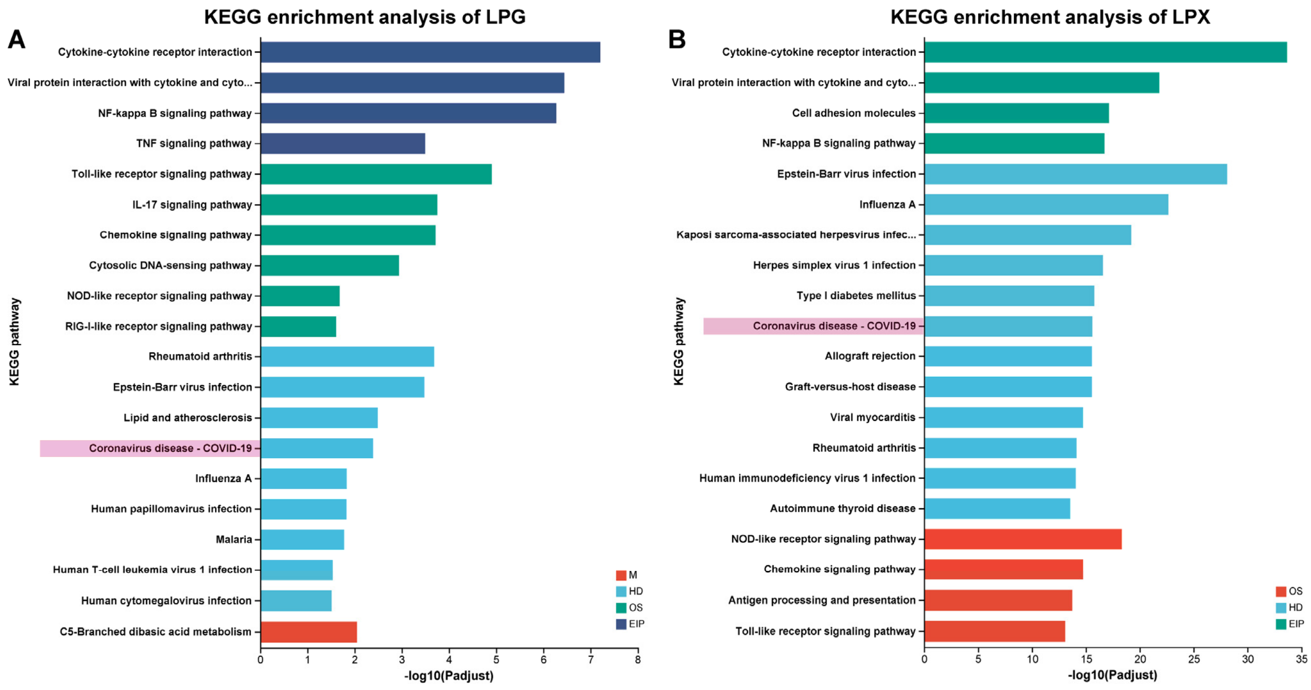

A
KEGG enrichment analysis of LPG
KEGG pathway
Cytokine-cytokine receptor interaction
Viral protein interaction with cytokine and cyto...
NF-kappa B signaling pathway
TNF signaling pathway
Toll-like receptor signaling pathway
IL-17 signaling pathway
Chemokine signaling pathway
Cytosolic DNA-sensing pathway
NOD-like receptor signaling pathway
RIG-I-like receptor signaling pathway
Rheumatoid arthritis
Epstein-Barr virus infection
Lipid and atherosclerosis
Coronavirus disease - COVID-19
Influenza A
Human papillomavirus infection
Malaria
Human T-cell leukemia virus 1 infection
Human cytomegalovirus infection
C5-Branched dibasic acid metabolism
M
HD
OS
EIP
0
1
2
3
4
5
6
7
8
-log10(Padjust)
B
KEGG enrichment analysis of LPX
KEGG pathway
Cytokine-cytokine receptor interaction
Viral protein interaction with cytokine and cyto...
Cell adhesion molecules
NF-kappa B signaling pathway
Epstein-Barr virus infection
Influenza A
Kaposi sarcoma-associated herpesvirus infec...
Herpes simplex virus 1 infection
Type I diabetes mellitus
Coronavirus disease - COVID-19
Allograft rejection
Graft-versus-host disease
Viral myocarditis
Rheumatoid arthritis
Human immunodeficiency virus 1 infection
Autoimmune thyroid disease
NOD-like receptor signaling pathway
Chemokine signaling pathway
Antigen processing and presentation
Toll-like receptor signaling pathway
OS
HD
EIP
0
5
10
15
20
25
30
35
-log10(Padjust)

Figure S3

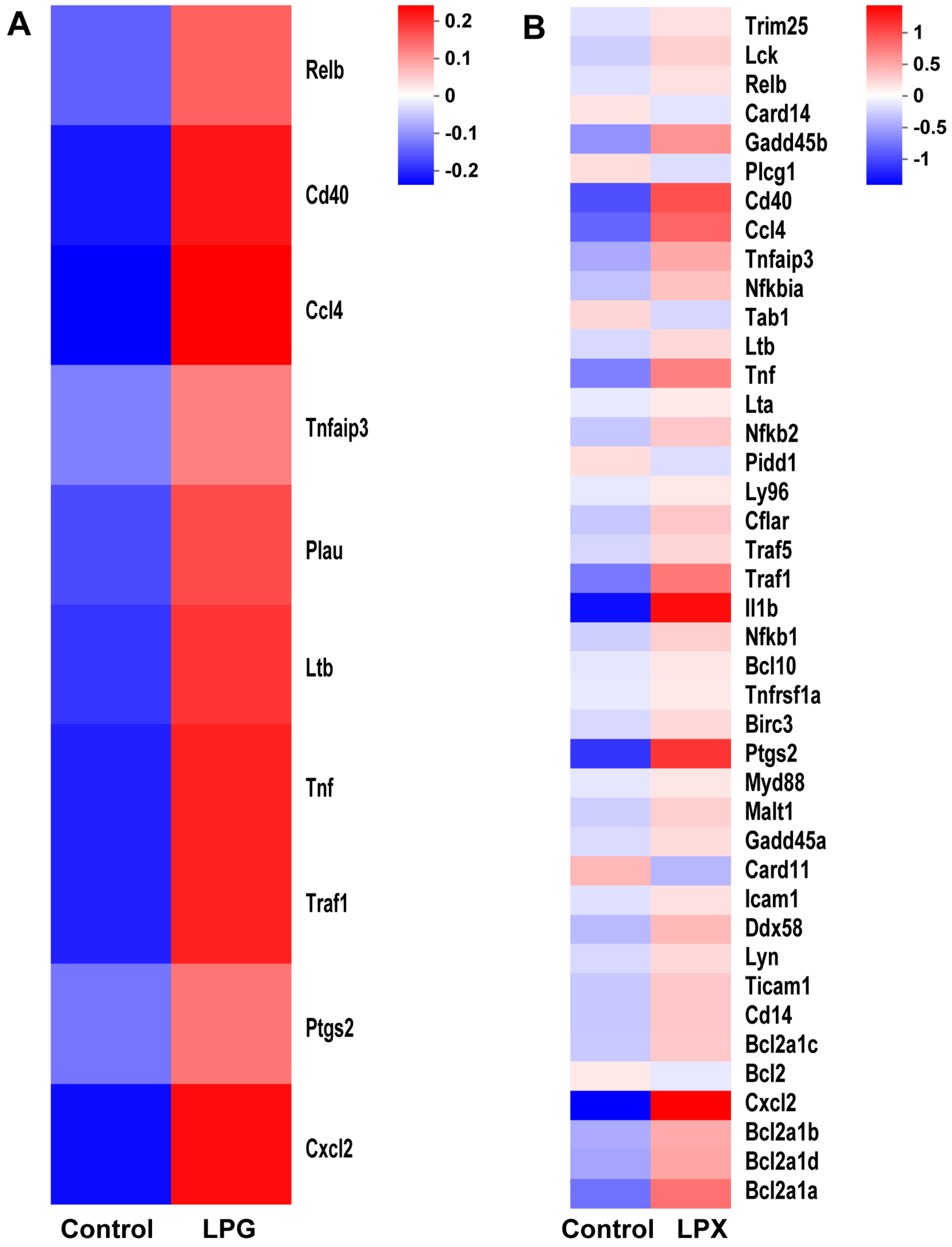
A
Relb
Cd40
Ccl4
Tnfaip3
Plau
Ltb
Tnf
Traf1
Ptgs2
Cxcl2
0.2
0.1
0
-0.1
-0.2
Control
LPG
B
Trim25
Lck
Relb
Card14
Gadd45b
Plcg1
Cd40
Ccl4
Tnfaip3
Nfkbia
Tab1
Ltb
Tnf
Lta
Nfkb2
Pidd1
Ly96
Cflar
Traf5
Traf1
Il1b
Nfkb1
Bcl10
Tnfrsf1a
Birc3
Ptgs2
Myd88
Malt1
Gadd45a
Card11
Icam1
Ddx58
Lyn
Ticam1
Cd14
Bcl2a1c
Bcl2
Cxcl2
Bcl2a1b
Bcl2a1d
Bcl2a1a
1
0.5
0
-0.5
-1
Control
LPX
